# Assessment of nasalance and nasality in patients with a repaired cleft palate

**DOI:** 10.1007/s00405-017-4506-y

**Published:** 2017-03-15

**Authors:** Klaus Sinko, Maike Gruber, Reinhold Jagsch, Imme Roesner, Arnulf Baumann, Arno Wutzl, Doris-Maria Denk-Linnert

**Affiliations:** 1 Department of Cranio-, Maxillofacial and Oral Surgery, Medical University, Waehringer Guertel 18-20, 1090 Vienna, Austria; 2 Faculty of Psychology, Institute of Clinical Psychology, University of Vienna, Vienna, Austria; 3 Division of Phonatrics-Logopedics, Department of Otorhinolaryngology, Medical University, Vienna, Austria

**Keywords:** Cleft palate, Nasality, Instrumental diagnosis, Nasalance, Sensitivity, Specificity

## Abstract

In patients with a repaired cleft palate, nasality is typically diagnosed by speech language pathologists. In addition, there are various instruments to objectively diagnose nasalance. To explore the potential of nasalance measurements after cleft palate repair by NasalView^®^, we correlated perceptual nasality and instrumentally measured nasalance of eight speech items and determined the relationship between sensitivity and specificity of the nasalance measures by receiver-operating characteristics (ROC) analyses and AUC (area under the curve) computation for each single test item and specific item groups. We recruited patients with a primarily repaired cleft palate receiving speech therapy during follow-up. During a single day visit, perceptive and instrumental assessments were obtained in 36 patients and analyzed. The individual perceptual nasality was assigned to one of four categories; the corresponding instrumental nasalance measures for the eight specific speech items were expressed on a metric scale (1–100). With reference to the perceptual diagnoses, we observed 3 nasal and one oral test item with high sensitivity. However, the specificity of the nasality indicating measures was rather low. The four best speech items with the highest sensitivity provided scores ranging from 96.43 to 100%, while the averaged sensitivity of all eight items was below 90%. We conclude that perceptive evaluation of nasality remains state of the art. For clinical follow-up, instrumental nasalance assessment can objectively document subtle changes by analysis of four speech items only. Further studies are warranted to determine the applicability of instrumental nasalance measures in the clinical routine, using discriminative items only.

## Introduction

A cleft lip and palate (CLP) is a relatively common congenital malformation, with an incidence of about 1 in 700 newborns in the Caucasian population [1]. While an isolated cleft lip primarily is an aesthetic problem, complete CLP may cause velopharyngeal insufficiency (VPI) which can interfere with speaking, breathing, and swallowing. Often CLP is associated with an articulation disorder generally regarded as compensation to an anatomical abnormity leading to VPI. This may lead to dysfunctions not only of the velopharyngeal sphincter, but also of the entire vocal tract [2]. CLP-associated VPI typically leads to deviations in the resonance such as hypernasality or hyponasality, nasal air emission and weak pressure consonants, and compensatory articulation [3].

VPI is an eminently clinical diagnosis. Any surgical intervention to correct the underlying anatomy should be planned, based on the combination of video-naso-pharyngoscopy and multiplanar videofluoroscopy. Magnetic resonance imaging is an emerging diagnostic tool, however, to date not widely adopted [4, 5].

Typically, speech and language pathologists assess articulation placement and manner, and examine the oral cavity and the pharynx through direct vision and palpation of the hard and soft palate [6]. However, the most important diagnostic procedure is a subjective evaluation by speech and language pathologists, who assess hyper-or hyponasality, nasal air emission and/or turbulances, consonant production errors, and voice disorders. For this subjective evaluation, the patients’ language background and age are important.

A more objective method may be the so-called nasometry, which measures nasalance instrumentally and can provide objective data for evaluating nasal resonance.

Different centers may use different speech parameters in testing, and therefore, they are not always comparable [7]. Several assessment protocols have been described [8–10], but none of them was widely adopted. The most accepted assessment protocol was developed by an international working group. Henningsson et al. [11] reported an universal system for reporting speech outcome measures. The system includes five universal characteristics like hypernasaly, hyponasality, nasal air emission and/or turbulances, consonant production errors, and voice disorders. Concerning the grading of hypernasality, mild forms are appreciated primarily in certain vowels and may not be socially disturbing to the patient or family. Moderate hypernasality is audible with most vowels and deemed socially unacceptable. Severe hypernasality would usually prompt a recommendation for intervention by the patient and the clinician, because speech intelligibility is significantly diminished [11]. A standard test regimen for the perceptual nasality evaluation is routinely performed using specific test items, like those from the Heidelberg Rhinophonia assessment form [12]. Typically, for perceptive nasality assessment, a scale with three to five grades is used. Recently, Baylis et al. [13] described that the use of analog visual scales is more accurate than the nasality documentation within a few categories only. Many studies investigate the use of instrumental objective diagnosis, which typically provide higher diagnostic resolutions compared to only few assessment classes diagnosed by speech therapists [1, 14–16].

Our goal was to determine possible differences between perceptual and instrumental measurements in the east Austrian area. We selected various items of the Heidelberg Rhinophonia assessment form and determined their nasalance scores on the NasalView^®^ System to explore their potential to assist the perceptive nasality assessment.

## Materials and methods

### Patients

We recruited 39 patients grown up in eastern Austria with a repaired cleft palate. All patients (or their parents) provided written informed consent to their study specific video/voice recording and instrumental nasalance assessment. All recruited patients consented to the electronic storage of their speech recordings, personal data, and the use of their assessments for scientific purposes. Because our study focused on hypernasality after cleft palate repair, we excluded three patients with obvious nasal obstructions, e.g., due to acute infections and in one case a Cul-de-sac resonance. There were no further inclusion criteria, such as age or gender.

### Perceptual nasality assessment

We selected four vowel items out of the Heidelberg Rhinophonia test form, two words with fricatives and plosives and an oral sentence (without any nasal consonants), and one sentence with eight nasal consonants (Table 1). We termed the four items without nasal contents (#1, #3, #6, #7) as “oral” and the items with nasal content as “nasal” (#2, #4, #5, #8).

**Table 1:** Selected 8 items from the Heidelberg Rhinophonia assessment form

	Speech item^a^ No.	Speech instruction	Comments
Vowels	1	aaa–aaa–aaa	/a/ repeated
	2	iii–iii–iii	/i/ repeated
	3	aaaaaaaa	/a/ sustained
	4	iiiiiiii	/i/ sustained
Words	5	kikeriki–kikeriki–kikeriki	/kikeriki/ repeated
	6	fasa–fasa–fasa	/fasa/ repeated
Sentences	7	Peter spielt auf der Weide	Oral sentence
	8	Nenne meine Mama Mimi	Nasal sentence

^a^Speech items taken from the modified Heidelberger Rhinophonie assessment form

During the testing, a video with sound of each patient was recorded. Two speech therapists (one experienced and one trainee) assessed the perceptive nasality of the patients. We provided both evaluators with the identical video records, to reduce the burden for the patients and also minimize any variance in the patients’ presentations. The evaluators assessment was categorized in 4 grades (grade 0—normal, 1—mild, 2—moderate, and 3—severe hypernasality) as proposed by an international working group [11].

### Instrumental nasalance evaluation

Nasalance was measured with the NasalView^®^-System (Version 1.2, Tiger Electronics DRS Inc., Seattle, WA, USA). The instrument was calibrated according to the producers’ instructions. The instrumental measurements and the speech recordings on the videos were done during the same appointment. For the comparison of the instrumental and the perceptive assessments, we used the same speech items for the NasalView^®^ measurements and the perceptive evaluation. For each of the eight speech items, the mean nasalance values per test person were statistically analyzed.

### Data processing and statistics

**Fig. 1:**
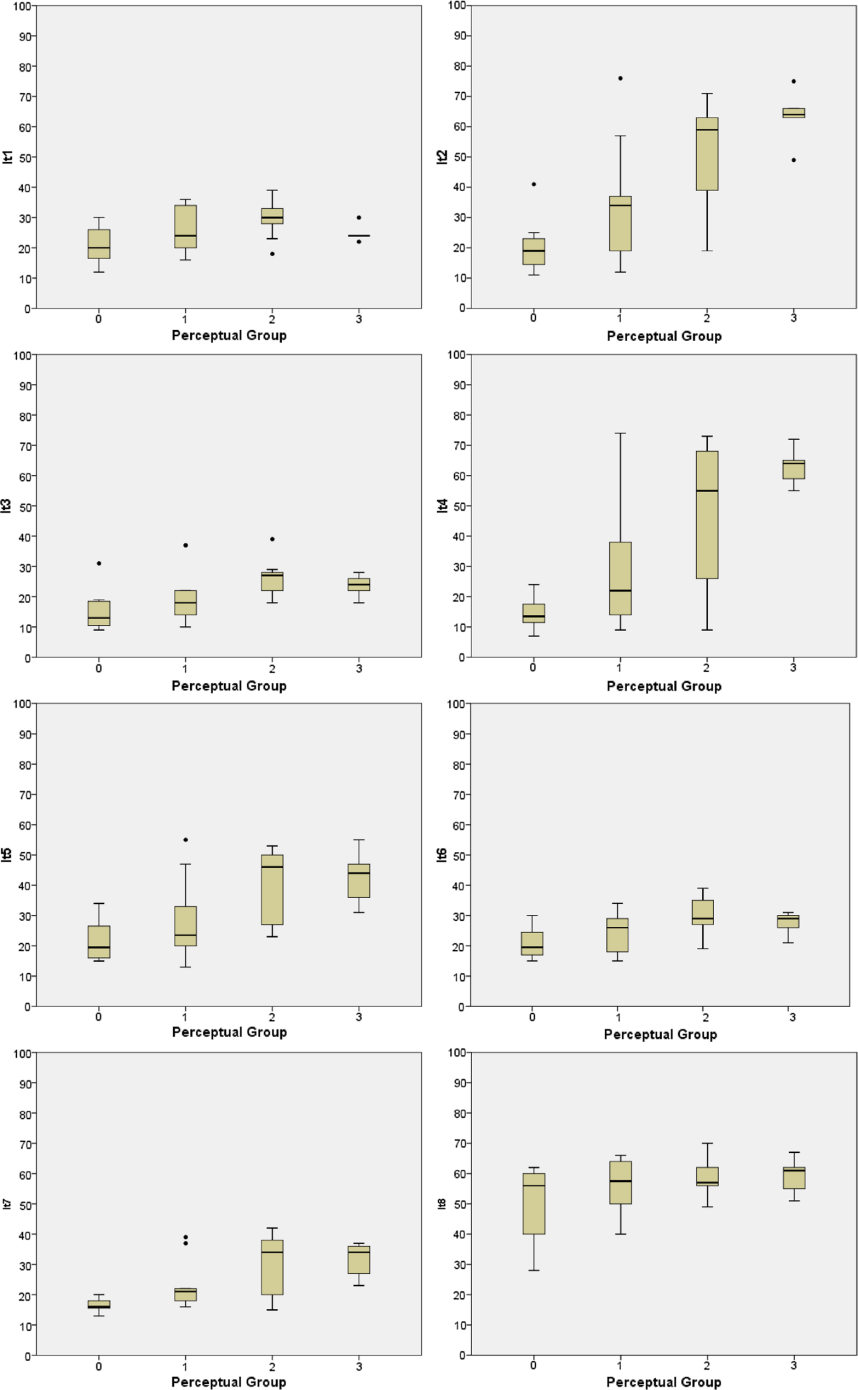
*Box plots* perceptual/instrumental. Plot of the four diagnostic nasalance groups against the NasalView^®^ results. *Boxplots* with medians, quartiles, minimum and maximum, and outliers of the eight speech items #1, #2, #3, #4, #5, #6, #7, #8

**Fig. 2:**
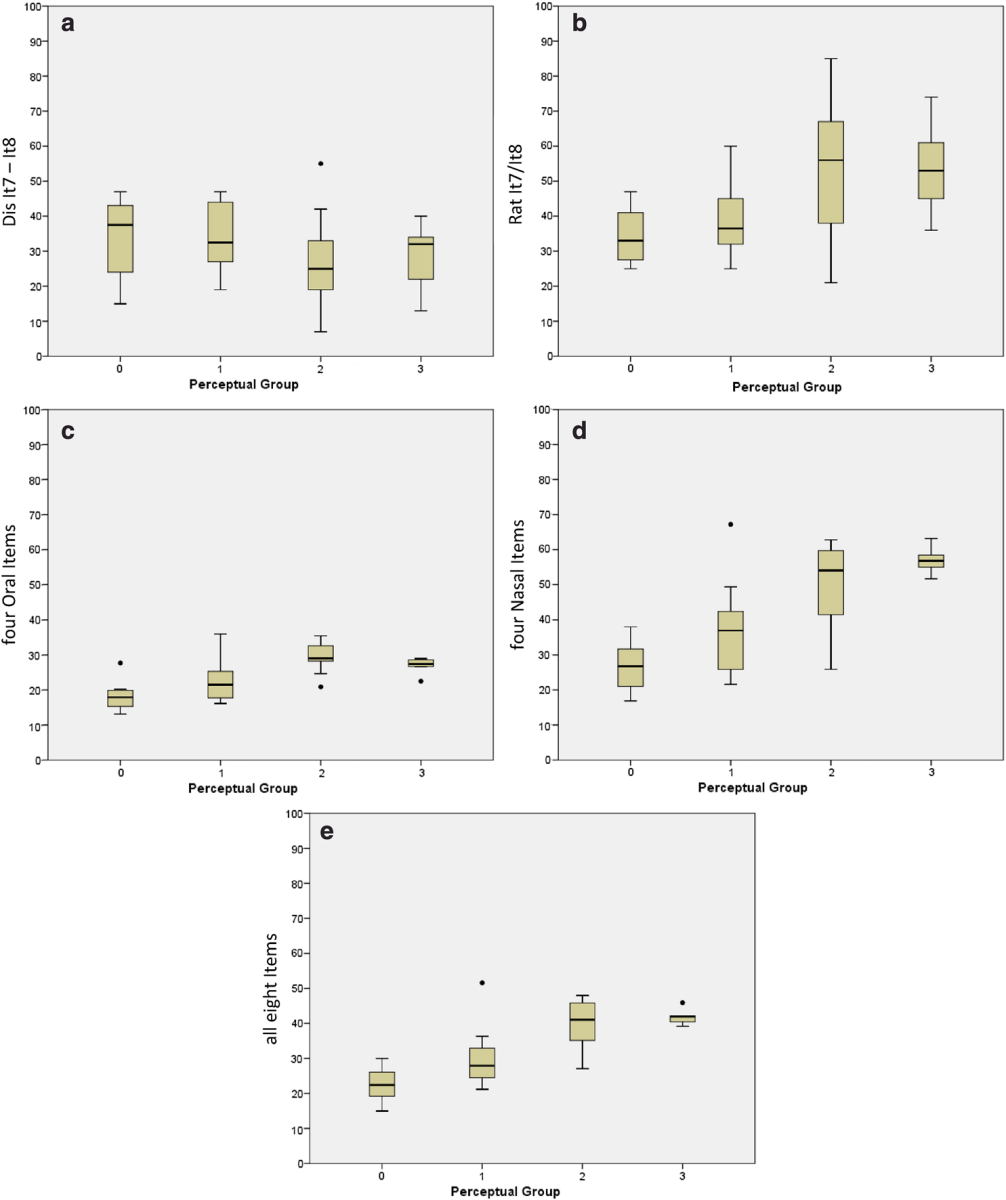
Grouped speech items. *Box plots* of the four diagnostic groups; median, quartiles, minimum and maximum, and outliers of ND (**a**) and NR (**b**) of the speech items #7 and #8; group score of oral speech items #1, #3, #6, and #7 (**c**); group score of nasal speech items #2, #4, #5, and #8 (**d**); and group score of all eight speech items (**e**)

For the statistical analyses, we used the computer program Stata (Version 13.1, StataCorp, TX, USA) to analyze mean and standard deviations. The interrater reliability between the two speech therapists was analyzed by computing Cohens kappa with linear weighting [17], and was found to match “almost perfect”. Although the analysis showed no difference between the novice and the experienced observer, just to avoid intermediate scores, we plotted the diagnostic groups of the experienced speech therapist against the NasalView^®^ measurements in box plots (Figs. 1, 2).

**Fig. 3:**
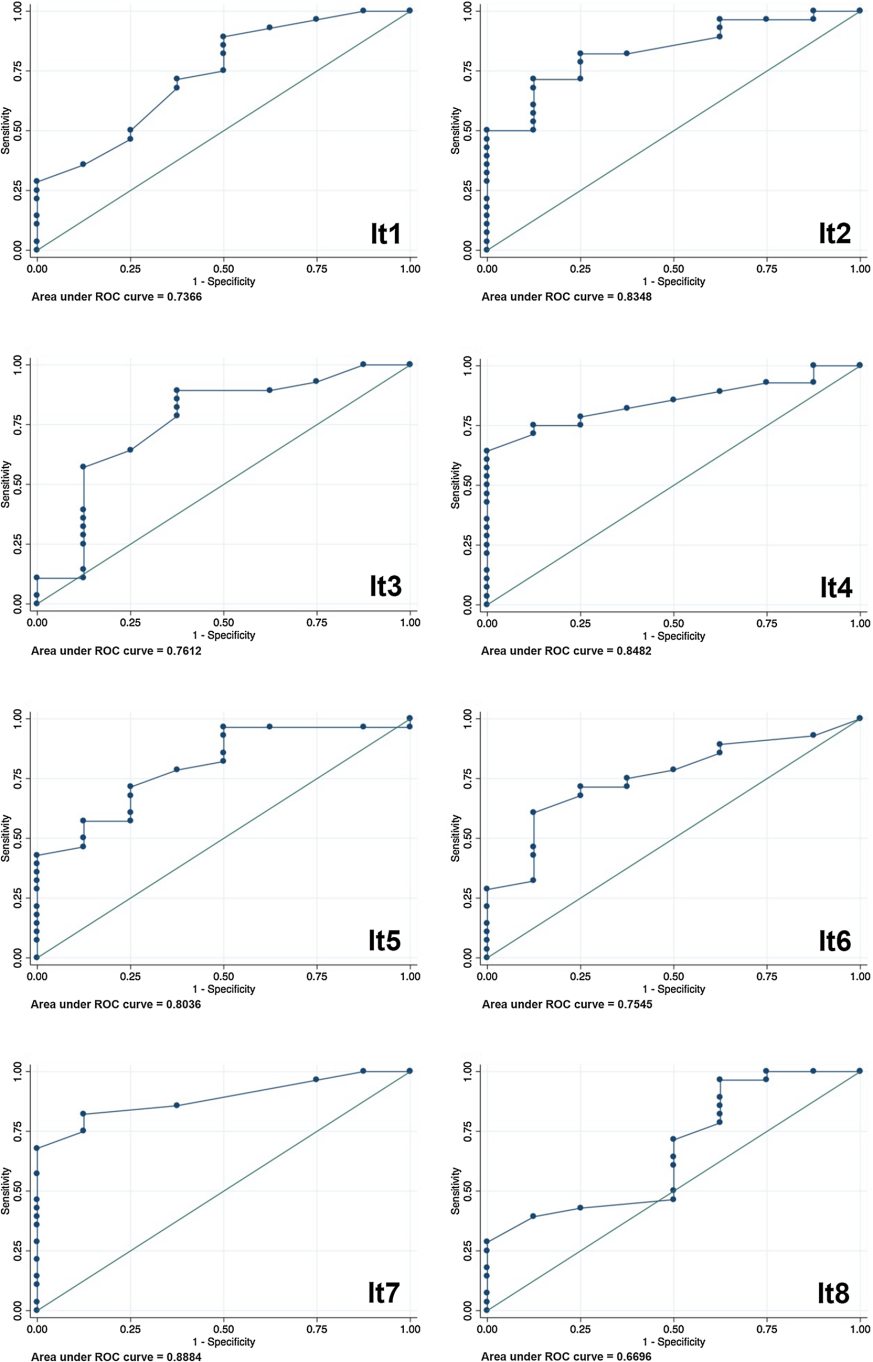
ROC curves, 8 items. Receiver-operating characteristics (ROC) to determine the accuracy of the test reliability of NasalView^®^ measurement compared to the perceptual nasality measurement. The ROC curves show the relationship between the perceptual and instrumental method; eight individual speech items #1, #2, #3, #4, #5, #6, #7, #8

**Fig. 4:**
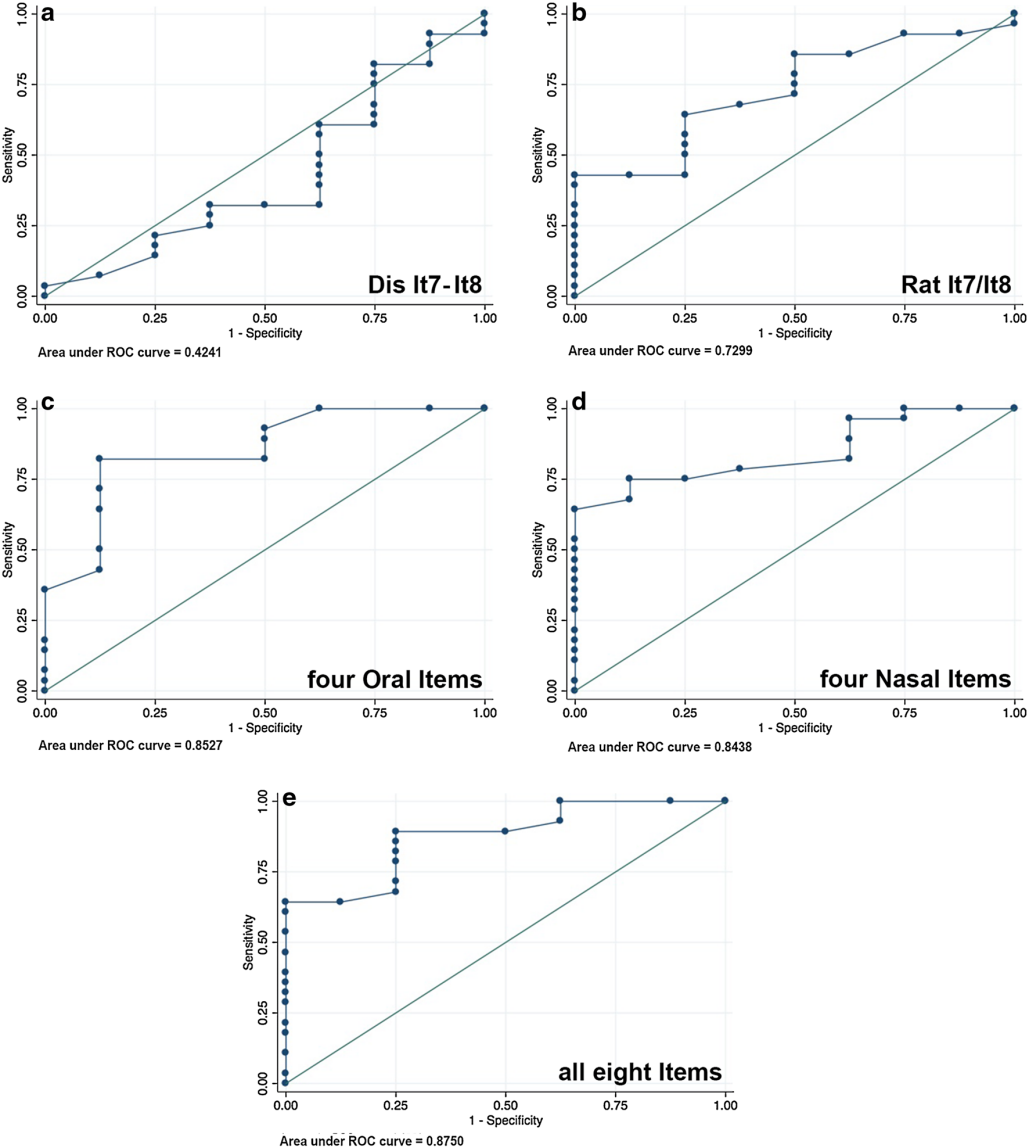
ROC, combined items. Receiver-operating characteristics (ROC) to determine the accuracy of the test reliability of NasalView^®^ measurement compared to the perceptual nasality measurement. The ROC curves show the relationship between the perceptual and instrumental method; ND (**a**) and NR (**b**) of the speech items #7 and #8; grouped oral speech items #1, #3, #6, and #7 (**c**); grouped nasal speech items #2, #4, #5, and #8 (**d**), and all eight speech items (**e**)

To discard suboptimal speech items, we tested the individual items and item groups by receiver-operating characteristics (ROC) analyses as done before by Bressmann [18]. Because ROC analyses are a binary classifier system [19], we transformed the ordinate data of the perceptive tests into two categories. The group rated “0” was determined “normal nasality”, the groups rated “1, 2, and 3,” were summarized to “hypernasality”. Figures 3 and 4 show the ROC curves.

In the next step to describe how well the test separates the groups with and without nasality, we measured the accuracy by the area under the ROC curve. The area under the curve is a measure of correctly classified nasality assessments. We computed the cutpoints and the areas under the curve (AUCs), and determined the sensitivity and specificity for each of the eight individual speech items and various speech item groups (Table 2).

**Table 2:** Correlation of subjective and objective speech evaluations

Item	Nasal/Oral	Cut point	Sensitivity (%)	Specificity (%)	Correctly classified (%)	AUC
1	O	19	89.29	50.00	80.56	0.7366
*2*	*N*	*16*	*96.43*	*37.50*	*83.33*	*0.8348*
3	O	14	89.29	62.50	83.33	0.7612
*4*	*N*	*9*	*100.00*	*12.50*	*80.56*	*0.8482*
*5*	*N*	*18*	*96.43*	*50.00*	*86.11*	*0.8036*
6	O	18	89.29	37.50	77.78	0.7545
*7*	*O*	*16*	*96.43*	*25.00*	*80.56*	*0.8884*
8	N	47	96.43	37.50	83.33	0.6696
ND 7 and 8	O–N	29	60.71	37.50	55.56	0.4241
NR 7 and 8	O/N	32	85.71	37.50	77.78	0.7299
Oral	4O	17	92.86	50.00	83.33	0.8527
Nasal	4N	25	96.43	37.50	83.33	0.8438
All	4N + 4O	24	89.29	75.00	86.11	0.8750

^a^Speech items with high AUC (from 0.8 to 0.89) and high sensitivity (≥96.43%) are recommended for use with instrumental nasality measures (#2, #4, #5, and #7) and printed in *italics*

## Results

### Participants

36 participants contributed to the data set for this study. The age range was from 8 to 27 years (15.4 ± 5.3 mean ± SD); 13 participants were female (36.1%) and 23 were male (63.9%). Most patients (86.1%) were CLP patients (*N* = 31; 11 female, 20 male). Five patients (13.9%; 3 female, 2 male) had an isolated cleft palate. Of the 31 CLP patients, the cleft was on the left side in 15 cases (48.4%), in 8 patients on the right (25.8%), and in 8 patients (25.8%) on both sides. In our study cohort, we observed no statistically differences with respect to gender, location, and type of the malformation (data not shown). 22.2% of the patients revealed a normal nasality and 77.8% of the patients were hypernasal.

### Interrater agreement

The interrater reliability between the two speech therapists resulted in a weighted kappa of 0.8816 (almost perfect match, *ĸ*_w_ = 0.81–1.0).

### Perceptive vs. instrumental diagnosis

The data obtained with the NasalView^®^-System were categorized based on the four perceptual assessment groups: group 0 (*N* = 8, normal), group 1 (*N* = 14, mild), group 2 (*N* = 9, moderate), and group 3, (*N* = 5, severe hypernasality). Figure 1 shows the instrumentally measured nasalance distributions for each perceptually diagnosed grade per single speech item. The nasal items #2, #4, and #5, and the oral item #7 showed the best linear correlation with the four perceptual grades (Fig. 1; Table 2). Figure 2 shows the results of nasal distance (ND) and the nasalance ratio (NR) from speech item #7 and #8, all four oral and all four nasal speech items, or all eight speech items together. Among all groups of four or eight items, only the nasal group revealed similar results as the four best single items (Fig. 2; Table 2).

Figure 3 shows the ROC analyses for each speech item. Figure 4 shows the results of ND and NR from the speech items #7 and #8, the average of the oral or the nasal items, or the average from all eight speech items. For the discrimination of specificity and sensitivity, the AUC and the content of correctly classified patients are listed in Table 2. Based on these two parameters, by taking into account the correctly classified patients and the AUC, we determined the cut points, and the respective sensitivity and specificity (Table 2).

The ND and NR of the speech item #7 and #8, as well as the oral (#1, #3, #6 and #7), and nasal (#2, #4, #5 and #8) speech items, as well as all eight speech items together did not reveal a higher sensitivity than the four single speech items #2, #4, #5, and #7 (Table 2).

## Discussion

By comparing the gold standard “perceptual diagnosis” with instrumental measurements, we found speech items, suitable for the instrumental follow-up assessments in patients with diagnosed hypernasality. The sensitivity of only four specific speech items is superior to the averaged sensitivity using additional speech items. Therefore, our findings may provide clinicians with a strategy to increase the sensitivity in the follow-up of patients with perceptively diagnosed nasality.

Considering the reliability of perceptually diagnosed nasality, multiple raters may be preferred compared to the diagnosis by only one evaluator [20]. Therefore, we engaged two speech therapists for the perceptive assessment: an experienced speech therapist and a trainee. It is clear that a comparison between two speech therapists cannot be statistically significance, and therefore, our specific results cannot be extrapolated to other clinical centers. The comparison of the vowel [i:] and [a:] reflects a possible compensatory function of the tongue. During [i:] phonation, the tongue is positioned high close to the soft palate. During phonation of the vowel [a:], the tongue lies deep and far back [21]. Our findings that the use of a nasal and oral sentence can provide clinically useful results confirm Bressmann [18], who described that the use of only one short nasal and one short oral sentence does not compromise the validity of the examination. In addition, Watterson, et al. [22] described that speech items with a minimum of six syllables are sufficient for valid determination of nasalance. Therefore, we selected a short sentence without nasal consonants (Table 1; item #7) and another with only nasal consonants (Table 1, item #8). To reveal possible additional information, we computed the so-called nasalance distance (ND) and nasalance ratio (NR) from the two sentences (item #7 and #8). The nasal sentence reveals the nasalance maximum, while the oral sentence indicates the individuals’ nasalance minimum [23].

When plotting the four perceptually diagnosed groups against the NasalView^®^ measures, we observed a good correlation with some but not all individual speech items. The positive discrimination was best in the speech items 2 and 4 (Fig. 1). The ND and NR between speech item #7 and #8 corresponded rather poorly to the perceptual grading (Fig. 2). The mean of grouped speech items (four oral, four nasal, all 8 speech items) discriminated rather well between the perceptual and instrumental measures (Fig. 2).

The comparison of perceptive and instrumental assessments is a common approach to investigate the theory and praxis of speech assessment [24]. Using ROC curves, we revealed that the nasalance associated with individual items and item groups corresponded at various degrees to the perceptual assessments.

NasalView^®^ measurements can correlate with perceptual assessments [16]. Single speech items can correlate better than item groups (Table 2). However, it would be a key flaw to adopt our specific results to other clinical centers without further tests, simply because a comparison of only one speech language pathologist with an instrumental diagnostic method cannot reveal statistically significant results.

Each of the single nasal speech items #2, #4, #5, and the oral speech item #7, provided the same or better AUC measures compared to the four oral, or the four nasal speech items combined, or all 8 speech items in total (compare Fig. 3, with Fig. 4). The computed sensitivity and specificity for each single item analysis of speech item #2, #4, #5, and #7 score highest for sensitivity (96.43–100%), while the averaged “four oral” “four nasal” or “all eight speech items together” scored lower (89.29–96.43%; Table 2). The specificity of all results was below acceptable standards (Table 2).

Our findings with the NasalView System^®^ are only partially transferable to other systems due to differences between instruments [25]. Because, in our study, the specificity of the instrumental nasalance measures was generally low, our findings support the previously published opinion, that instrumental assessment can never substitute, but only complement perceptual evaluation [7].

Socio-cultural and regional slang affects the comparability between studies [26, 27]. Seaver et al. [28] and Watterson et al. [29] considered regional differences and proposed the need for standardization for different regions. The usability of specific speech items may depend on the cultural and linguistic background of the assessed person [30].

Nasalance can vary between individual speakers and regional dialects [26]. However, in the follow-up situation where every patient provides his/her personal baseline and only intraindividual comparisons are relevant, a repeated instrumental assessment can document subtle changes. Therefore, we postulate that the instrumental assessment can be used independently of the patients´ specific linguistic background.

Bressmann et al. [23] described the ND and the NR as useful values, which can provide additional nasalance information. In our study, the sensitivity and specificity of ND and NR between speech item #7 and #8 were low (Table 2; AUC < 0.73). However, ND and NR may depend on the individual test person and specific test items.

Instrumental measures could be superior to perceptual examination in two aspects: finer scale (0–100%, instead of 0–3 in perceptual assessments) and objectivity of the instrument. As Baylis et al. [13] described that the use of a finer scale can provide more accurate documentation, instruments may provide better opportunities to quantitatively describe subtle improvements during follow-ups after therapeutic interventions.

## Conclusion

The perceptual assessment of nasality by speech language pathologists remains the gold standard method for diagnosis, as it can also elucidate the grade of speech impairment. Instrumental evaluation cannot replace perceptual examination. However, after hypernasality has been diagnosed by perceptual methods, the instrumental nasalance assessment—due to the finer scale—may provide objectively documented subtle changes in the follow-up evaluation. Further studies to test the efficacy of instrumentally assisted follow-ups (e.g., after surgical intervention) are warranted.

## References

[1] Peterka M, Peterkova R, Tvrdek M, Kuderova J, Likovsky Z (2000). Significant differences in the incidence of orofacial clefts in fifty-two Czech districts between 1983 and 1997. Acta Chir Plast.

[2] McWilliams B, Morris H, Shelton R (1990) Cleft palate speech. B. C. Decker, Toronto

[3] Golding-Kushner KJ (2001) Therapy techniques for cleft palate speech and related disorders. Singular 10.1597/1545-1569_2004_41_340_ltte_2.0.co_2 15151439

[4] Sinko K, Czerny C, Jagsch R, Baumann A, Kulinna-Cosentini C (2015). Dynamic 1.5-T vs 3-T true fast imaging with steady-state precession (trueFISP)-MRI sequences for assessment of velopharyngeal function. Dentomaxillofac Radiol.

[5] Fu M, Barlaz MS, Holtrop JL, Perry JL, Kuehn DP, Shosted RK, Liang ZP, Sutton BP (2016) High-frame-rate full-vocal-tract 3D dynamic speech imaging. Magn Reson Med 10.1002/mrm.26248 27099178

[6] Ysunza PA, Repetto GM, Pamplona MC, Calderon JF, Shaheen K, Chaiyasate K and Rontal M (2015) Current controversies in diagnosis and management of cleft palate and velopharyngeal insufficiency. BioMed Res Int 196240 10.1155/2015/196240 PMC4529889 26273595

[7] Peterson-Falzone SJ, Hardin-Jones MA, Karnell MP (2010). Assessment of speech language problems in Cleft Palate Speech 4edn.

[8] John A, Sell D, Sweeney T, Harding-Bell A, Williams A (2006). The cleft audit protocol for speech-augmented: a validated and reliable measure for auditing cleft speech. Cleft Palate Craniofac J.

[9] Grunwell P, Brondsted K, Henningsson G, Jansonius K, Karling J, Meijer M, Ording U, Wyatt R, Vermeij-Zieverink E, Sell D (2000). A six-centre international study of the outcome of treatment in patients with clefts of the lip and palate: the results of a cross-linguistic investigation of cleft palate speech. Scand J Plastic Reconstruct Surg Hand Surg.

[10] Sell D, Harding A, Grunwell P (1999). GOS.SP.ASS.’98: an assessment for speech disorders associated with cleft palate and/or velopharyngeal dysfunction (revised). Int J Lang Commun Disord Royal College of Speech Lang Therap.

[11] Henningsson G, Kuehn DP, Sell D, Sweeney T, Trost-Cardamone JE, Whitehill TL (2008). Universal parameters for reporting speech outcomes in individuals with cleft palate. Cleft Palate Craniofac J.

[12] Hirschberg J, Gross M (2006). Velopharyngeale Insuffizienz mit und ohne Gaumenspalte.

[13] Baylis A, Chapman K, Whitehill TL and Group TA (2015). Validity and reliability of visual analog scaling for assessment of Hypernasality and audible nasal emission in children with repaired cleft palate. Cleft Palate Craniofac J.

[14] Kozéluh SWK, Joos U (2005) Objektive Diagnostik von Hypernasalität bei LKGS-Patienten mit dem NasalView-System. Forum Logopädie

[15] Karling J, Larson O, Leanderson R, Galyas K, de Serpa-Leitao A (1993). NORAM—an instrument used in the assessment of hypernasality: a clinical investigation. Cleft Palate Craniofac J.

[16] Melzer F, Otten JE, Dittmann J, Löhle E (2007) Nasalance—und Nasalitätsmessungen bei Patienten mit Spaltfehlbildungen Sprache Stimme Gehör 31:84–89

[17] Landis JR, Koch GG (1977). The measurement of observer agreement for categorical data. Biometrics.

[18] Bressmann T (1999) Sprechsprachliche und psychosoziale Aspekte bei Patienten mit Lippen-Kiefer-Gaumenspalten, Untersuchungen zu nasaler Resonanz, Sprechgeschwindigkeit, Stimmklang und Lebensqualität. Inaugural Dissertation, Ludwigs- Maximilians-Universität

[19] Begg CB (1987). Biases in the assessment of diagnostic tests. Stat Med.

[20] Kuehn DP, Moller KT (2000). Speech and language issues in the cleft palate population: the state of the art. Cleft Palate Craniofac J.

[21] Gildersleeve-Neumann CE, Dalston RM (2001). Nasalance scores in noncleft individuals: why not zero?. Cleft Palate Craniofac J.

[22] Watterson T, Lewis KE, Foley-Homan N (1999). Effect of stimulus length on Nasalance scores. The Cleft Palate-Craniofac J.

[23] Bressmann T, Sader R, Whitehill TL, Awan SN, Zeilhofer HF, Horch HH (2000). Nasalance distance and ratio: two new measures. Cleft Palate Craniofac J.

[24] Brancamp TU, Lewis KE, Watterson T (2010). The relationship between nasalance scores and nasality ratings obtained with equal appearing interval and direct magnitude estimation scaling methods. Cleft Palate Craniofac J.

[25] Bressmann T (2005). Comparison of nasalance scores obtained with the Nasometer, the NasalView, and the OroNasal System. Cleft Palate Craniofac J.

[26] Mueller R, Beleites T, Hloucal U, Kuehn M (2000). Objektive Messung der normalen Nasalanz im sächsischen Sprachraum. HNO.

[27] Hirschberg J, Bok S, Juhasz M, Trenovszki Z, Votisky P, Hirschberg A (2006). Adaptation of nasometry to Hungarian language and experiences with its clinical application. Int J Pediatr Otorhinolaryngol.

[28] Seaver EJ, Dalston RM, Leeper HA, Adams LE (1991). A study of nasometric values for normal nasal resonance. J Speech Hear Res.

[29] Watterson T, Lewis KE, Murdock T and Cordero KN (2013) Reliability and validity of nasality ratings between a monolingual and bilingual listener for speech samples from English-Spanish-Speaking children. Folia phoniatrica et logopaedica : official organ of the International Association of Logopedics and Phoniatrics (IALP) 65:91–97 10.1159/000353809 24157638

[30] Mayo R, Floyd LA, Warren DW, Dalston RM, Mayo CM (1996). Nasalance and nasal area values: cross-racial study. Cleft Palate Craniofac J.

